# A diagnostic tool for basic daily quality assurance of a tomotherapy Hi•Art machine

**DOI:** 10.1120/jacmp.v10i4.2972

**Published:** 2009-10-15

**Authors:** Iwein Van de Vondel, Koen Tournel, Dirk Verellen, Michael Duchateau, Steven Lelie, Guy Storme

**Affiliations:** ^1^ Department of Radiotherapy Oncologic Centre UZ Brussel Belgium; ^2^ XIOS Hogeschool Limburg Belgium

**Keywords:** helical tomotherapy, dose calibration, energy, quality assurance

## Abstract

To investigate and evaluate the use of an in‐house developed diagnostic software tool using the imaging detector data for a quick daily quality assurance check of the output (dose) and lateral profile (cone) of a tomotherapy Hi•Art system. The Hi•Art treatment system is a radiation therapy machine for delivering intensity modulated radiation therapy (IMRT) in a helical fashion with an integrated CT scanner used for improved patient positioning before treatment. Since the system was developed specifically for IMRT, fat fields can be obtained by modulating the beam and therefore the fattening filter could be omitted. Because of this, the field has a cone‐like profile in both lateral and transversal directions. Patients are treated in a helical fashion with a tight pitch and a constant gantry rotation speed, while modulation is performed by a binary MLC. Consequently dose output per time‐unit (dose rate) as well as the shape of the cone‐profile are very important for correct patient treatment and should be closely monitored. However, using the company‐provided initial tools and conventional dosimetry, this can be a time consuming daily procedure. The aim of this work is to develop a fast, automated method of quality assurance based on the detector signal. A software tool called “tomocheck” running on the operation station has been developed to evaluate the output (dose rate) and the lateral cone profile (energy) of the Hi•Art system, comparing actual output and cone profile with a reference (previously approved against ionization chamber measurements). This is done by using the data of the 640 on‐board detector array that are directly retrieved and processed after a specific QA procedure. The detector file consists of the CT detector data and the three monitoring dose chamber readings over a time period of 200 sec. To evaluate the method, the system was benchmarked against ionization chamber measurements and classical IMRT QA methods. Action levels (final status “NOT ACCEPTED”) for dose ratio as well as the cone ratio are set to ±2%. The QA tool was introduced for daily QA in May 2007. For the following 24 months, a total of 931 morning checks was made on both tomotherapy machines. In 42 cases the check status was “NOT ACCEPTED”. In 34 cases the dose ratio (DR) was out of tolerance. The corrected cone ratio (CCR) was outside of specification tolerance in 8 cases. The tomocheck data was related to the ionization chamber measurements for the IMRT plan indicating a close relationship between the CCR and the off‐axis measurements. Average dose ratio against the mean value of the on‐ and off‐axis IC measurement indicates that this parameter is a good interpretation of the dose output. This tool makes it possible to perform an easy‐to‐use and fast basic daily quality assurance check featuring an output as well as an energy evaluation. Ideally this tool should offer also the combined dosimetry check of jaw width, couch speed, leaf latency, output, leaf/gantry synchrony, and lasers. This will be investigated in the future.

PACS 87.55Qr

## I. INTRODUCTION

In July 2005 we installed as one of the first hospitals in Europe a Helical Tomotherapy radiation therapy unit (TomoTherapy Inc., Madison, WI, USA). One year later a second unit was installed. The Hi•Art treatment system is a radiation therapy machine for delivering intensity‐modulated radiation therapy (IMRT) in a helical fashion with an integrated CT scanner used for correct patient positioning before treatment. As the system was developed specifically for IMRT, fat fields can be obtained by modulating the beam and therefore the fattening filter could be omitted. Because of this, the field has a cone‐like profile in the both lateral and transversal directions.

Patients are treated in a helical fashion with a tight pitch and a constant gantry rotation speed, while modulation is performed by a binary MLC. Consequently, dose output per time‐unit (dose rate), as well as the shape of the cone‐profile, are critical for correct patient treatment and should be closely monitored. Daily output check of radiation therapy delivery equipment is one of the main components of any quality assurance (QA) program. The AAPM TG‐40 recommends that the output of a megavoltage radiation unit has to be checked every morning and assigns a ±3% acceptability window.^(1)^

The difference in the output requirements between a tomotherapy unit and the standard linear accelerator is that the output per time‐unit (dose rate) should be constant for the tomotherapy unit, while this is less important for the standard linear accelerator. The ability to deliver the planned dose‐distributions by a helical tomotherapy machine depends on four factors:^(2)^ static beam dosimetry (i.e. output and cone profile), system dynamics, system synchrony, and system geometry.

According to recommendations of Fenwick et al.,^(2)^ the daily checks of a helical tomotherapy measurement should include:

1. Output constancy quick check
2. TPR20/10 quick check
3. Lateral profile constancy quick check
4. Output ramp‐up time
5. Combined dosimetry check of jaw width, couch speed, leaf latency, output, and leaf/gantry synchrony
6. Lasers

Our first goal was to check only a part of the beam dosimetry: the output (characterized as dose‐rate) and the axis profile in the lateral direction. These checks are normally performed by utilizing ionization chambers placed in a solid water phantom (output) and film or water tank measurements (cone profile). Since these procedures require a long setup and processing time, it is not possible to do them every morning or during a treatment day without interfering with patient treatments.

Considering the overall tomotherapy radiation system, we decided to investigate the use of the available detector data acquired following the standard procedure to perform the daily quality assurance

## II. MATERIALS AND METHODS

### A. Tomotherapy unit and QA procedure

The Tomotherapy Hi•Art system consists of four basic components: the planning station, the optimization server, the data server, and the Radiation Delivery Subsystem (RDS).

The RDS includes all hardware and software for scan and treatment procedures of a patient.

At the core of RDS lays a ring gantry‐mounted short linear accelerator which generates X‐rays that are collimated into a fan beam using a binary multileaf collimator to modulate the intensity with the gantry angle. The RDS software components are responsible for reading, translating, and transferring data throughout the delivery subsystem. Its major components are the Data Acquisition System (DAS), the Data Receiver Server (DRS), the On‐Board Computer (OBC), and the Stationary Computer (STC). The detector used in the tomotherapy system is an arc‐shaped CT detector array.

The detector array consists of 738 cells filled with xenon with a 0.73 mm width at isocenter, and each cell is comprised of two gas cavities that are divided by thin tungsten septal plates 2.54 cm long in the beam direction. The irradiation sequence introduced for this QA procedure is a rotational treatment with a gantry speed of 3 rotations per minute, all leafs open, the jaws set to 5 cm, and the couch out of the bore. This “rotational variation” procedure is delivered to create a detector file that is then compared against the gold standard detector file previously approved against ionization chamber measurements.

### B. Detector data format

The entire detector file covers a wide range of data from which we isolated the MVCT detector data and the three dose monitoring chambers data. The MVCT detector reads the amount of exit radiation as the beam passes through the patient and the couch. Because the rotational variation procedure used in this test is without patient or couch in the beam (couch movement is inhibited and out of the bore), the detector channels’ readout is correlated with the lateral profile.

The MVCT detectors are composed of ionization chambers; the mean value of all detectors is an independent indication of the dose output. The default dose output is obtained by the readings of three monitoring dose ionization chambers. During the procedure, every 3.3 msec, a dump of all relevant data is saved on one of the internal computer systems (OBC, STC and DAS). After each treatment procedure, all the parameters are assembled and averaged out by a factor of 10 in the detector data file and transferred to the central Data Receiver Server (DRS). With a “rotational variation” procedure of 200 seconds a detector file of 6000 records will be generated, each record contains 640 detector channel data and the dose data of the three ionization chambers.

### C. The tomo diagnostic tool

#### C.1 System overview

The software graphical user interface (GUI), called “tomocheck”, is an in‐house development and is written in Visual C++ 2005 (Microsoft Corporation, Washington, USA) and runs on the operation station of the TomoTherapy Hi•Art system. The check program itself, the log file and the reference data file (gold standard) are all installed on a removable hard disk, avoiding possible interference with the tomotherapy software. Only an initialization file is installed on the hard disk.

The transfer of the detector data stored on the Data Receiver Server and the operation station is automatically performed using the File Transfer Protocol (FTP).

#### C.2 Working procedure

While delivering the QA procedure described earlier, the detector reads the amount of exit radiation and stores it on one of the internal computer systems. In addition, the dose counts measured with the ionization chambers are stored.

After the treatment has been delivered, the Hi•Art software gives an indication that the detector data is transferred to the data server. When started, the tomocheck program automatically performs a File Transfer Protocol (FTP) command to transfer the detector data file stored on the DRS to the operation station. After complete acquisition of the detector file, the algorithm cross‐correlates the dose data with the golden standard dose data, the MVCT detector data with the golden standard MVCT data, and the ratio of the dose data with the average of the MVCT detector data to the same ratio obtained from the golden standard.

#### C.3 The tomocheck program

The program uses an initialization file, which is a text based file and contains all settings and tolerances related to the QA procedures. It is stored on the hard drive of the operation station as it is linked to the machine by the machine number.

One of these parameters is the size of the detector file and this is set to 6000 records. This is a check to see if the correct Quality Assurance procedure is chosen.

Tolerances for dose‐ and cone‐ratios are set as follows:

- ‘Accepted’ (green) if lower than 1%
- ‘Accepted’ (orange) if ratio is between 1% and 2% (warning level)
- ‘Not accepted’ (red) if exceeding 2% (action level)

Tolerance for detector dose ratios is set green if lower than 2% and red if higher.

If the appropriate ratio (cone or dose) exceeds the tolerance, the check button will be shown in the corresponding color. If a detector channel differs more then 20% from the mean of the neighboring channels, the corresponding channel will be considered as malfunctioning.

If a dose output differs 10% from the readings before and after this reading, the system considers this as arcing. Many arcs will be indicated by the software via an “ACCEPTED BUT NEED ATTENTION” window.

When calculating the cone ratio, the edges are ignored by using the detector channels 70 to 570.

**Figure 1:**
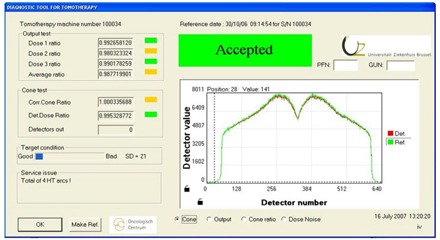
The program layout of the tomocheck program. Five separate areas can be identified: output test, cone test, target condition, service issues, and the profile view area. The cone view is selected here. This profile is the average cone profile obtained by the detector during the whole treatment procedure together with the average cone profile of the reference.

Figure 1 shows the program layout of the tomocheck program. It features five areas.

In the top left corner, the tomotherapy machine number is displayed. In the top right panel, the date and the hour of acceptance of the reference and the corresponding machine number are displayed. If the machine number saved with the reference doesn't match the machine number saved in the initialization file, an error is displayed and no ratios can be calculated. Only the current detector profile is displayed without the reference profile. In the right bottom corner, the date and time of the measurements are displayed. The “Output Test” pane shows the three dose ratios and the average dose ratio. The data from the three monitoring dose chambers are extracted from the detector file and can be represented^(3)^ as:

(1) $$DC_{i}=\left[d_{1}d_{2}\cdot \cdot \cdot d_{n}\right]$$

In this formula: 1 is ≤i≤3, which accounts for the three monitoring dose chambers and n=6000 (a quality procedure of 200 seconds generates a detector file of 6000 records). The average dose counts (ADC) can then be calculated with

(2) $$\begin{array}{lll} ADC_{i}=\frac{1}{n}\sum_{j=1}^{n}DC_{ij} & \; & 1\leq i\leq 3\;\;,\;\;n=6000 \end{array}$$

We calculate the average of the dose chambers’ counts to filter out the periodic variation of the dose output that exists due to synchrony between linac pulsing and rotation.

With the ADC, the dose ratio for each chamber is calculated by dividing this calculated value with the value that is stored in the reference file:

(3) $$DR_{i}\frac{{\left[ADC_{i}\right]}_{\text{cur}\text{ren}t}}{{\left[ADC_{i}\right]}_{\text{ref}\text{ere}\text{nce}}}\;\;\;\;\;\;1\leq i\leq 3$$

To filter out possible small differences between dose readout of the different dose chambers, a more robust dose readout is accomplished by calculating the average of the three dose ratios (DR):

(4) $$\bar{DR}=\frac{1}{3}\sum_{i=1}^{3}DR_{i}$$

If the appropriate ratio exceeds the tolerance, the check button will be shown in the corresponding color (orange or red). If it is within the tolerance, a green button will be shown.

The “Cone Test” pane displays the Corrected Cone Ratio.

The calculation of the cone ratio uses the MVCT detector array data. In contrast to the one‐dimensional data array of the monitoring dose chambers, the detector array is a two‐dimensional matrix which contains m×n elements (m=640 detectors, n=6000 records).

(5) $$\text{Det}\text{ect}or\;\text{Arr}ay=\left[\begin{array}{cccc} a_{11} & a_{12} & \ldots & a_{1n}\\ a_{21} & a_{22} & \ldots & a_{2n}\\ \vdots & \vdots & \ddots & \vdots \\ a_{m1} & a_{m2} & \ldots & a_{mn} \end{array}\right]$$

This data matrix is compressed into a column vector to filter out small fluctuations in the dose rate and arcing. This is written by

(6) $$b\;j=\sum_{i=1}^{6000}a_{ij}\;\;\;\;\;\;\;\;1\leq j\leq 640$$

In this formula, $b_{j}$ is the cumulated dose count received by each detector channel during the complete irradiation.

Note that the response of each detector is different due to the machine geometry. The fact that the curvature radius of the detector is not the same as the one of the machine itself will give another response and, therefore, a lower cumulated dose count at the middle detectors for the same dose.

The cone ratio, CR, is defined as the ratio between measurement and reference, and is calculated for each channel using

(7) $$CR\;j=\frac{{\left[b\;j\right]}_{\text{cur}\text{ren}t}}{{\left[b\;j\right]}_{\text{ref}\text{ere}\text{nce}}}\;\;\;\;\;\;\;\;1\leq j\leq 640$$

Then the average cone is calculated to obtain one single parameter that can be used to represent the cone deviation:

(8) $$CR_{\text{glo}\text{bal}}=\frac{1}{640}\sum_{j=1}^{640}CR\;j$$

To ignore the side effects, the system ignores the first and last 70 detector channels by using only the detector channels between channel 70 and 570 to calculate the cone ratio.

(9) $$CR=\frac{1}{500}\sum_{j=70}^{570}CR\;j$$

In order to be able to separate output effects and profile effects, the signal of the cone has to be corrected for the machine output. This is easily done by dividing the CR by the DR. The new parameter is called the corrected cone ratio.

(10) $$\text{CCR}=\frac{CR}{\bar{DR}}$$

The “Detector Dose Ratio” is a double ratio. First, determine the ratio between the dose monitoring chamber 1 and the average of the detector channels.

(11) $$\underset{\text{rat}io=}{}\sum_{i=1}^{6000}ADC_{1i}/{\;}_{\left(\frac{1}{640}\sum_{j=1}^{640}b_{j}\right)}$$

The DDR is calculated by taking the ratio between the measurement and the reference:

(12) $$\text{DDR}=\frac{\text{rat}io_{\text{cur}\text{ren}t}}{\text{rat}io_{\text{ref}\text{ere}\text{nce}}}$$

The system displays also the number of malfunctioning detectors. If a detector channel value (out of the 640 channels) differs more than 20% from the mean of the neighboring channels, the corresponding channel will be considered as broken.

The criterion can be written as:

(13) $$b\;j〈\left(\frac{b_{j-5}\;+\;b_{j-4}\;+\;K\;+\;b_{j+4}\;+\;b_{j+5}}{10}\right).0.80$$

The variable $b_{j}$ represents the dose counts of they $j^{\text{th}}$ channel.

**Figure 2:**
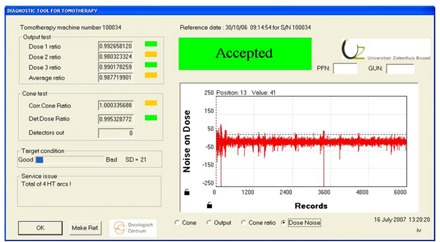
The noise signal on the dose 1 chamber. Target issues can be predicted or identified here. An increased noise signal indicates a worse target condition (possible delaminating of layers or a hole burned in the target).

The Target condition bar (graphical indication) gives an indication of the condition of the target observed from the dose chamber signals. When zooming in on the signal, what looks like “grass growing on a hill” can be an indication that the target is not uniform as it turns around (Fig. 2). There could be layers of the target delaminating or a hole burned through it. Consequently, the noise on the dose chamber signal can be used as a relative measure for the target condition as well as to identify target issues. A bigger noise signal indicates a worse target condition. The system calculates the standard deviation of the noise signal to have a numeric value for the target condition. This value appears in the Target condition pane.

The “service issue” message window shows the number of arcs and identifies possible malfunctioning detectors. An arc is identified if a dose value (internal ionization chamber) drops more than 10% compared with the overall dose value. The right side of the window shows the selected signal profile. One can select cone, output, cone ratio, and dose noise view. Zoom features are provided.

The cone view (Fig. 1) shows the average cone profile during the whole treatment procedure together with the average cone profile of the reference. In ideal circumstances, these two profiles are identical. Note the dip in the center of the signal, which is an artifact caused by the fact that the focus of the detector does not coincide with the center of rotation.

**Figure 3:**
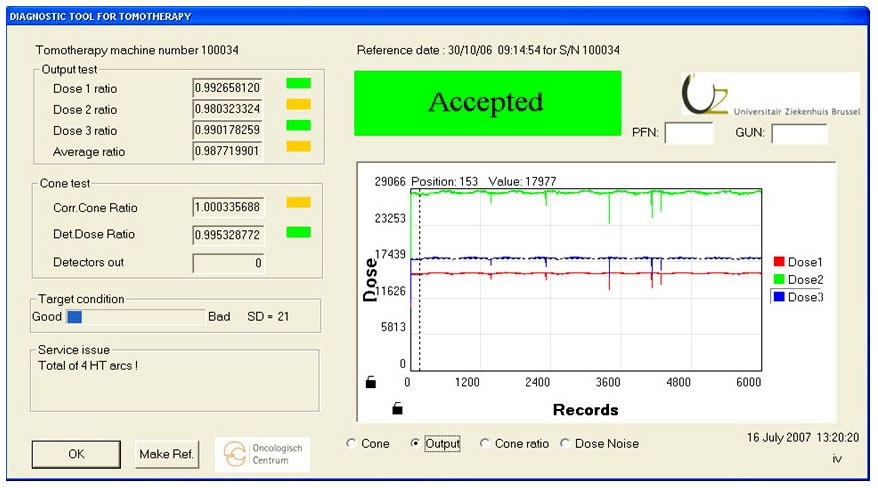
The output view. The signals of the three ionization chambers are displayed as a function of the time expressed in records. With a “rotational variation” procedure of 200 sec, 6000 records will be generated, containing the dose date of the three ionization chambers.

The output is visualized by the display of the signals of the three ionization chambers as a function of the records acquired during the irradiation procedure (Fig. 3). As such this can be an indication of variation in dose output. Only the current dose signals are displayed. To calculate the doses ratios, the average value of the corresponding dose chamber is divided by the average value of the reference dose value. Arcing issues can be evaluated here. Many drop pulses in the dose signal are an indication of arcing. The latter can be caused by contaminated waveguide gas (SF6), the magnetron or the linac. The system counts the arcs and displays the number in the service window. If the number of arcs exceeds ten (parameter entered in the initialization file and experimentally found as a good threshold), a warning message “Excessive arcing” will be displayed.

**Figure 4:**
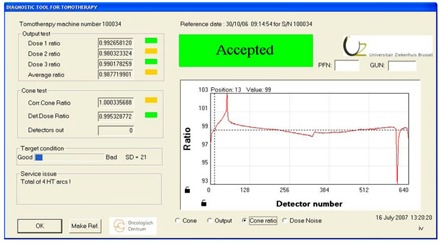
The cone ratio view – a graphical interpretation of the difference in current lateral cone profile with the reference cone profile. To have a good numerical value of the cone ratio, the outer lateral detectors have to be ignored.

The cone ratio (Fig. 4) is a graphical interpretation of the difference in current cone profile with the reference cone profile. To have a good numerical value of the cone ratio the outer lateral detectors shows artifacts and have to be ignored. That is the reason why only the detectors mentioned in the initialization file are taken into account for calculating the cone ratio.

Depending on the calculations of the ratios, a “global acceptance icon” will be shown.

If one of the ratios is out of tolerance (red), the global icon will display “NOT ACCEPTED” in red. If all ratios are within tolerance (green or orange), the icon will display ACCEPTED in green. If there is a service problem (such as excessive arcing or bad detectors), the icon will also be green and display the message “ACCEPTED BUT NEED ATTENTION”. If the NOT ACCEPTED icon shows, it is an indication that the output or cone is not correct. This has to be investigated and can be solved by changing the PFN Voltage (pulse forming network) or the GUN current (injector). Once these corrections are performed, the tomocheck procedure has to be repeated. Before closing this window, the new parameters (PFN V and GUN I) can be entered in the edit windows at the right pane in the check program and will be saved together with the ratios and other relevant data in a log file.

If there is no reference file present, the system displays only the current cone profile, the output view, and the dose noise. No ratios can be calculated.

To assign the current detector file as the new reference file (most likely after full dosimetrical QA), one can select the “Make Ref” button. A password is required to confirm the new reference. The system offers also the possibility to browse for reference data acquired at a previous date.

#### C.4 Logging features

The tomocheck program keeps a log of all ratios and relevant data.

After evaluating the output and cone profile of the tomotherapy machine, the following variables will be saved for statistics: the current date and time, reference date and time, the three dose readings, the three reference dose readings, dose ratios, cone ratio, dose/detector signal ratio, broken detectors, number of arcs, dose noise, service messages if any, current tolerances (copied from the initialization file), the entered PFN Voltage and/or Gun current, and the final status of the “global acceptance icon”.

All these parameters can be evaluated later with a spreadsheet program.

#### C.5 Validation

The validation can be subdivided in two stages. The first stage is evaluating the system using static beam measurements. However, this static beam does not reflect a typical treatment setup. Therefore, a second stage of rotational beam measurements was performed.

The first stage demonstrates the direct link between the tomocheck variables and the machine output. Data are acquired at different dose rates for a static beam and are compared with ionization chamber (IC) measurements at depth of maximum dose in a solid water phantom for a 5 cm by 40 cm field. The dose rate was altered gradually by changing the PFN. The output of the three monitoring dose chambers, as well as the corrected cone signal, was plotted against the IC measurements to investigate linearity, consistency, and traceability.

**Figure 5:**
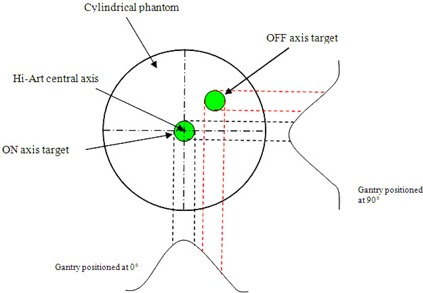
An illustration of the importance of the ON and OFF axis targets. The first on‐axis target volume will only be treated by the center part of the cone profile; the second target volume is located off‐axis and the dose in the center of this volume will be composed by different regions of the cone profile representing a combined effect of output and cone shape.

In the second stage, we used a rotational quality assurance procedure irradiating two target volumes in a cylindrical phantom. With a tomotherapy procedure, the patient is treated in a helical way while moving inside the gantry. Because of the special design of the machine, the rotational isocenter (where the static measurement is performed) is not necessarily located in the center of the tumor, as is the case on a conventional machine. This implies that the dose in a given point is again a combination of output, cone shape, and MLC modulation. At our clinic, these parameters are verified using a generic IMRT plan on a cylindrical phantom (GAMMEX‐RMI, Middleton, US). This planned phantom includes two cylindrical volumes receiving different doses during treatment. These cylindrical volumes are situated on‐axis and off‐axis, meaning that one target is situated on the axis of rotation and the other isn't. The first on‐axis target volume will only be treated by the center part of the cone profile, with only minor leaf modulation present. The second target volume is located off‐axis and the dose in the center of this volume will be composed by different regions of the cone profile representing a combined effect of output and cone shape (Fig. 5). Performing daily ionization chamber measurements on these phantom treatments for 2.5 cm and 5 cm field widths allowed us to check consistency of output and cone profile for both field sizes, and is a good measure of the daily performance of the system. However, these measurements are also time consuming, taking about 50 minutes to complete.

To investigate if the tomocheck program has enough predictive power to replace the daily measurements, 34 IMRT ionization chamber measurements, taken on different days, for the 5 cm and 2.5 cm field plan were performed on two target volumes irradiated with 1 and 2Gy, respectively, on‐ and off‐axis. Consecutively a tomocheck procedure was performed. Because the rotational variation procedure used in the tomocheck procedure is without patient or couch in the beam treatment couch, the cylindrical phantom was retracted after each IC measurement.

The correlation between the different tomocheck parameters and the IMRT measurements was investigated.

#### C.6 Software availability

We are well aware of the fact that the QA tool provides only basic checks for a quite complex radiation technique^(^ ^4^ ^,^ ^5^ ^)^. Therefore, it will be necessary to develop a combined dosimetry check of jaw width, couch speed, leaf latency, output, leaf/gantry synchrony, and lasers. This software tool is still in development and therefore not available for use by other tomotherapy users. Suggestions or collaborations with others users are always welcome and can be addressed to the author.

## III. RESULTS & DISCUSSION

The QA tool was installed on both tomotherapy machines in May 2007.

Every morning prior to the patient treatment program the physicist or engineer uses the software tool to evaluate the output and cone profile of each machine

**Figure 6:**
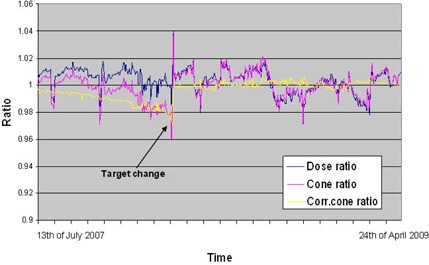
The dose ratio, cone ratio, and the corrected cone ratio for the second machine during 22 months. Because the dose and the cone ratios follow each other, we decided to correct the cone ratio with the dose ratio. This value is more representative of the differences between the reference and the current lateral profile.

The dose ratio can be interpreted as a measure for absolute output of the linear accelerator, where the cone ratio can be related to the ratio between the on‐ and off‐axis dose as measured with a phantom. The reason why the “Detector/Dose Ratio” is calculated as well is to check independently the dose chambers and the detector chambers. If one of those chambers fails, it would be indicated by this ratio. As of this writing, a total of 476 morning checks have been performed on unit 1, and 455 on unit 2. For machine 1, in 27 cases the final check status was “NOT ACCEPTED”. In 25 cases this was caused by an out of tolerance of the output (dose ratio); twice it was caused by an out of spec of the cone profile or both. For the second unit, the check indicated a red status in 15 cases: 9 caused by a dose ratio, and 6 by the cone ratio or both. A global “Not Accepted” was never caused by a bad “Detector/Dose Ratio”. The dose ratio, cone ratio, and the corrected cone ratio for the second machine for the 22 months are displayed in Fig. 6. Because the dose and the cone ratios are correlated, we decided to correct the cone ratio with the dose ratio in an attempt to filter output effects from cone effects. This value is more representative of the differences between the reference and the current lateral profile. When the dose or corrected cone ratio exceeded 0.98% or 1.02%, adjustment of the PFN value (magnetron current pulse) was made to adjust the dose within specification. The decrease of the corrected cone ratio with time is caused by deterioration of the target. We noticed also that a decreasing corrected cone ratio (lower than 0.98) is a more reliable indication of target condition than the calculated “target condition value” because the latter is influenced by magnetron‐arcing, linac arcing, GUN issues, and temperature.

The calculated target condition is a reliable indication only at the last months of the life of the target and if there were no arcs in the quality procedure.

To evaluate the three dose monitoring chambers data versus an external ionization chamber for different dose rates, variations in PFN values have been introduced.

**Figure 7:**
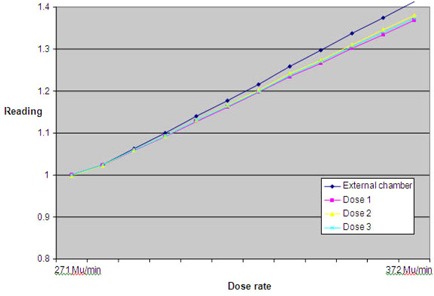
The PFN values have been varied to evaluate the data of the three dose monitoring chambers versus an external ionization chamber for different dose rates. The dose monitoring values, the reading from the external ionization chamber, and the detector profile were all measured with a dose rate varying from 271 MU/min to 372 MU/min.

The dose monitoring values, the reading from the external ionization chamber, and the detector profile were all measured with a dose rate varying from 271 MU/min to 372 MU/min (Fig. 7). Both detection methods show a linear relationship with the dose rate. However, the differences in chamber volume between external IC and monitor chambers, combined with the fact that not only the dose tempo changes but also the lateral profile with varying PFN, cause the relative dose chamber signal to have a smaller slope. The results show that the dose chamber signals provide an unambiguous tool for monitoring relative output changes.

The gold standard for output QA in our center are measurements performed on a generic IMRT plan on a symmetric phantom containing disjunctive target volumes on and off the linac rotation axis. The relationship between the tomocheck tool and these measurements was checked by comparing IMRT measurements to consecutively acquired tomocheck variables on 34 days.

**Figure 8:**
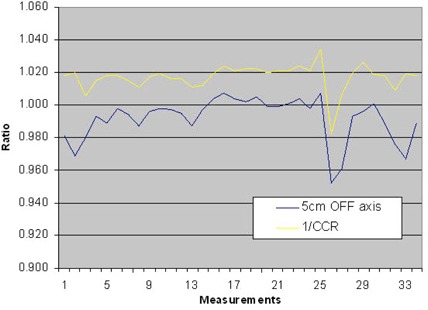
The Corrected Cone Ratio obtained from the tomocheck program versus the off‐axis IC measurement during 34 measurements performed on different days. The IC response is somewhat higher compared to the 1/CCR, which is indicated in the average ration of IC and 1/CCR equal to 0.97503. A SD of 0.00915 indicates the close relationship between both detection methods.

Figure 8 shows the corrected cone ratio obtained from the tomocheck program versus the off‐axis IC measurement during 34 measurements performed on different days. The IC response is somewhat higher compared to the 1/CCR, which is indicated in the average ration of IC and 1/CCR equal to 0.97503. A SD of 0.00915 indicates the close relationship between both detection methods.

**Figure 9:**
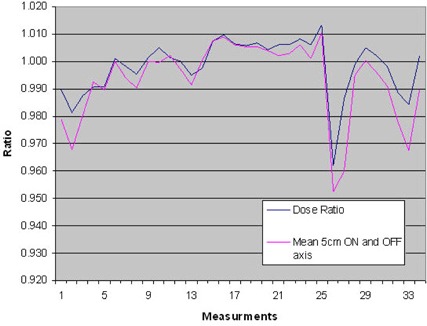
Average Dose Ratio against the mean value of the on‐ and off‐axis IC measurement again indicates that the tomocheck parameter is a good interpretation of the dose output (average ratio of DR and the IC measurements is 1.00506 with a SD of 0.00608).

Figure 9 shows average dose ratio against the mean value of the on‐ and off‐axis IC measurement again indicates that the tomocheck parameter is a good interpretation of the dose output (average ratio of DR and the IC measurements is 1.00506 with a SD of 0.00608).

## IV. CONCLUSIONS

The quality assurance of a Tomotherapy Hi•Art system is very complex and time consuming. This makes it very important to develop a new QA procedure that reduces the QA time.

The presented software tool makes it possible to perform an easy‐to‐use and fast basic daily quality assurance check featuring both output as well as an energy evaluation. This tool could be used to replace the typically‐used static QA and the generic plan QA. Another very important point is the tomo diagnostic log file which contains all tomocheck data over time. The evolution gives information about system related issues, such as target condition, by looking at the corrected cone ratio over a longer time period as well as other problems (such as magnetron malfunctioning that can cause increasing arcing counts).
